# Quality metrics in solid organ transplantation: protocol for a systematic scoping review

**DOI:** 10.1186/s13643-016-0279-4

**Published:** 2016-06-14

**Authors:** Kendra E. Brett, Alexandria Bennett, Nicholas Fergusson, Greg A. Knoll

**Affiliations:** Clinical Epidemiology Program, Ottawa Hospital Research Institute, 1967 Riverside Drive, Ottawa, K1H 7W9 Ontario Canada; Department of Medicine, University of Ottawa, Ottawa, Ontario Canada; Division of Nephrology, Kidney Research Centre, Ottawa Hospital Research Institute, Ottawa, Canada

**Keywords:** Solid organ transplantation, Quality, Health indicators

## Abstract

**Background:**

Transplantation is often the best, if not the only treatment for end-stage organ failure; however, the quality metrics for determining whether a transplant program is delivering safe, high quality care remains unknown. The purpose of this study is to identify and describe quality indicators or metrics in patients who have received a solid organ transplant.

**Methods/design:**

We will conduct a systematic scoping review to evaluate and describe quality indicators or metrics in patients who have received a solid organ transplant. We will search MEDLINE, Embase, and the Cochrane Central Register for Controlled Trials. Two reviewers will conduct all screening and data extraction independently. The articles will be categorized according to the six domains of quality, and the metrics will be appraised using criteria for a good quality measure.

**Discussion:**

The results of this review will guide the development, selection, and validation of appropriate quality metrics necessary to drive quality improvement in transplantation.

**Systematic review registration:**

PROSPERO CRD42016035353.

**Electronic supplementary material:**

The online version of this article (doi:10.1186/s13643-016-0279-4) contains supplementary material, which is available to authorized users.

## Background

Transplantation is the only treatment for some types of end-stage organ failure (e.g., liver) while it is the treatment of choice for others (e.g., kidney). In the case of kidney failure, transplantation is preferred since it improves quality of life, prolongs survival, and is less costly compared to dialysis [1–3]. From the perspective of the patient and the healthcare system, however, major problems remain in transplantation including lack of appropriate access; premature transplant failure and death; and reduced quality of life due to various complications.

The best approach for determining whether a transplant program is delivering high-quality, safe care remains unknown [4–6]. The USA has instituted mandatory reporting of survival data from all transplant hospitals [4]; however, this metric on its own may not adequately capture all aspects of transplant program quality as “poor” performance may lead to reduced access to transplantation services [4, 7, 8]. Healthcare quality encompasses important dimensions beyond effectiveness outcomes (e.g., graft survival), such as access to treatment, patient-centeredness, safety, and equity [9]. In transplantation, these aspects of quality are less well known. The purpose of this study is to systematically identify and review all quality metrics/indicators that have been used in the practice of solid organ transplantation.

## Research objectives

The aim of this study is to systematically review the literature in order to identify and describe the indicators/metrics that are used to measure the quality of care provided to solid organ transplant recipients. Our objectives are the following: first, to describe which quality metrics are being reported and how they are being used in solid organ transplantation; second, to categorize these metrics within the domains of quality [9]; and third, to evaluate whether these metrics fit the criteria for a good measure [10].

## Methods

### Search strategy

A comprehensive electronic literature search will be conducted in MEDLINE, Embase, and the Cochrane Central Register for Controlled Trials (CENTRAL) from inception to April 2016. The references of the included studies and existing reviews will be scanned for additional studies that were not identified by the search. The search strategy will be developed with the assistance of a medical librarian experienced in systematic reviews. A structured search strategy will be based on controlled vocabulary and relevant key terms, and will be broad to prioritize sensitivity (Additional file 1). Key search terms will include “quality”, “quality indicator”, “quality improvement” and ”quality of health care”. The language of publication will be restricted to the English language. This review has been registered with PROSPERO (CRD42016035353).

### Study screening and inclusion

Literature search results will be uploaded to EndNote X7 software, which will be used to find and remove duplicates. References will be sorted alphabetically by title and exported to Microsoft Word for screening. Two independent reviewers will screen the titles and abstracts (stage 1) against the inclusion criteria. Additional duplicates that may be missed by the referencing software will be identified during the screening process. For all titles and abstracts that appear potentially eligible, we will obtain full-text reports. If no abstract is available for a given citation, then the full-text will be obtained unless the article can be confidently excluded by its title alone. Two independent reviewers will screen all full-text reports to determine whether they meet the inclusion criteria (stage 2). Prior to the formal screening process, the two reviewers will participate in a short pilot exercise to identify and address any inconsistencies in the application of the screening criteria. A third reviewer will reconcile any disagreements between the two reviewers regarding an article’s inclusion status. The study selection process will be summarized using a PRISMA flow diagram [11].

### Study eligibility criteria

Studies will be selected according to predefined criteria. Peer reviewed, published articles will be included if they evaluate, measure, or review quality metrics/indicators in solid organ transplantation. Quality metrics are defined as “any objective measure that has been developed to support self-assessment and quality improvement at the provider, hospital and/or health care system” [12]. To be included, the metrics/indicators must be capable of some degree of quantification [13] and related to the quality of care that is provided to the transplant recipients by a transplant program. All metrics (e.g., structure, process, and outcomes) will be considered [6]. Metrics will be excluded for the following reasons: (i) they are not capable of some degree of quantification, (ii) the metric is dependent on non-modifiable factors, (iii) the metric is related to the organ donor or the organ procurement process, and (iv) the metric is used as a predictive tool, rather than an assessment of quality. We will include studies examining humans of all ages who have undergone a solid organ transplant (kidney, heart, liver, lung, pancreas, small bowel, and combinations of these organs). We will exclude studies involving bone marrow and cell transplants. There will be no restrictions on the design of the study, the type of setting, or the length of follow-up for outcomes.

### Data extraction

Data will be extracted from each eligible study by two independent reviewers using a pre-designed form in Microsoft Excel. The form will be pilot tested to ensure it captures all the relevant information. Any discrepancies that cannot be resolved by discussion will be adjudicated by a third reviewer.

Information pertaining to study identification (author, year of publication, number and location of centers, funding, and journal), study design (type of study, sample size, eligibility criteria, and length of follow-up), aggregate patient characteristics (age, gender, organ(s) studied, length of time since transplant, and comorbidities), and relevant quality metrics (name of metric(s), type of metric, measurement time points, the comparator outcome, main findings, and limitations of the metric) will be extracted.

### Data synthesis

We will use the PRISMA statement to guide the reporting of our findings. A completed copy of the PRISMA-P checklist is included as an additional file (Additional file 2). For all included studies, we will provide a detailed description of the quality metrics in both tables and text. The data will be organized based on organ type and population [adult (≥18 years) vs. pediatric (<18 years)]. The metrics will be categorized into the six domains of quality: access, equitable, patient-centered, efficiency, safety, and effectiveness (described in Table 1) [9, 14]. The quality metrics will be appraised using Janakiraman and Eker’s [10] criteria for a good measure: (i) easy to define and observe; (ii) important to patients and healthcare providers; (iii) amenable to change; and (iv) obtainable from existing or easily collected data. This appraisal will be presented as a table (Additional file 3), with each of the criteria receiving a score of “yes”, “no”, or “unclear”. This tool will not be used to calculate a score for each quality metric, but rather to present whether each metric meets some or all of the criteria for a good measure. It is expected that the data will be very heterogeneous, due to variations in the organs studied, the populations, the quality metrics, and the comparator measures/outcomes; therefore, no statistical analysis is planned. Metrics meeting all of the criteria for a good measure will be identified, and a narrative synthesis will be used to compare and describe how the metrics are being used within each quality domain.

**Table 1:** Domains of quality of health care [9, 14]

Domain of quality	Definition
Access	Delivery of health care that is timely, geographically reasonable, and provided in a setting where skills and resources are appropriate
Equitable	Delivering health care which does not vary in quality because of personal characteristics such as gender, race, ethnicity, or socio-economic status
Patient-centered	Delivering health care which takes into account the individual patient preferences, needs, and values
Efficiency	Delivering health care in a manner which maximizes resources use and avoids waste, including waste of equipment, supplies, ideas and energy
Safety	Delivering health care which minimizes risks and avoids injuries to patients from the care that is intended to help them
Effectiveness	Delivering health care that is adherent to an evidence base and results in improved health outcomes for patients, and refraining from providing services to those not likely to benefit.

## Discussion

The aim of this systematic scoping review is to evaluate and describe quality metrics that are used in solid organ transplantation. The rigorous and systematic nature of our review will ensure that it captures all relevant information on quality metrics in organ transplantation. Due to the multiple domains of quality, we have purposely created a relatively broad search strategy and study inclusion criteria in order to capture the most comprehensive look at quality metrics that are being used or have been proposed in solid organ transplantation. Reliable and valid quality metrics are necessary to establish baseline performance, to determine areas that need improvement, and to determine if practice changes have led to progress. The results of this study will identify innovative transplant quality indicators, which will help to guide the selection of core quality metrics which are suitable for further study, and can be used in subsequent transplant quality improvement initiatives that will actively measure and promote improvements in health outcomes.

## Abbreviations

CENTRAL, Cochrane Central Register for Controlled Trials; PRISMA, Preferred Reporting Items for Systematic Reviews and Meta-Analyses; PRISMA-P, Preferred Reporting Items for Systematic review and Meta-Analysis Protocols

## Additional files

Additional file 1: Search strategies. The data provided shows the comprehensive search strategy for each database. (DOCX 15.5 kb) Additional file 2: PRISMA-P checklist. The data provided shows a completed copy of the PRISMA-P checklist to guide readers in assessing the quality of the current protocol. (DOCX 39.5 kb) Additional file 3: The appraisal of candidate measures using the criteria for a good measure. The data provided demonstrates the tool that will be used to determine if a quality metric meets the criteria for a good measure. (DOCX 18.6 kb)

## References

[1] Laupacis A, Keown P, Pus N, Krueger H, Ferguson B, Wong C, Muirhead N (1996). A study of the quality of life and cost-utility of renal transplantation. Kidney Int.

[2] Tonelli M, Wiebe N, Knoll G, Bello A, Browne S, Jadhav D, Klarenbach S, Gill J (2011). Systematic review: kidney transplantation compared with dialysis in clinically relevant outcomes. Am J Transplant.

[3] Wong G, Howard K, Chapman JR, Chadban S, Cross N, Tong A, Webster AC, Craig JC (2012). Comparative survival and economic benefits of deceased donor kidney transplantation and dialysis in people with varying ages and co-morbidities. PLoS One.

[4] Kasiske BL, McBride MA, Cornell DL, Gaston RS, Henry ML, Irwin FD, AK, Metzler NW, Murphy KW, Reed AI, et al. Report of a consensus conference on transplant program quality and surveillance. Am J Transplant. 2012;12:1988–96. 10.1111/j.1600-6143.2012.04130.x 22682114

[5] Toussaint ND, McMahon LP, Dowling G, Soding J, Safe M, Knight R, Fair K, Linehan L, Walker RG, Power DA (2015). Implementation of renal key performance indicators: promoting improved clinical practice. Nephrology (Carlton).

[6] van der Veer SN, van Biesen W, Couchoud C, Tomson CR, Jager KJ (2014). Measuring the quality of renal care: things to keep in mind when selecting and using quality indicators. Nephrol Dial Transplant.

[7] Schold JD, Arrington CJ, Levine G (2010). Significant alterations in reported clinical practice associated with increased oversight of organ transplant center performance. Prog Transplant.

[8] Schold JD, Buccini LD, Srinivas TR, Srinivas RT, Poggio ED, Flechner SM, Soria C, Segev DL, Fung J, Goldfarb DA (2013). The association of center performance evaluations and kidney transplant volume in the United States. Am J Transplant.

[9] Institute of Medicine (2001). Crossing the quality chasm: a new health system for the 21st century.

[10] Janakiraman V, Ecker J (2010). Quality in obstetric care: measuring what matters. Obstet Gynecol.

[11] Moher D, Liberati A, Tetzlaff J, Altman DG (2010). Preferred reporting items for systematic reviews and meta-analyses: the PRISMA statement. Int J Surg.

[12] Bonow RO, Masoudi FA, Rumsfeld JS, Delong E, Estes NA, Goff DC, Grady K, Green LA, Loth AR, Peterson ED (2008). ACC/AHA classification of care metrics: performance measures and quality metrics: a report of the American College of Cardiology/American Heart Association Task Force on Performance Measures. J Am Coll Cardiol.

[13] Donabedian A (1988). The quality of care how can it be assessed. JAMA.

[14] World Health Organization (2006). Quality of care: a process for making strategic choices in health systems.

