# Supplementary material for: Quality metrics in solid organ transplantation: protocol for a systematic scoping review
Source: Syst Rev. 2016 Jun 14;5:99. doi: 10.1186/s13643-016-0279-4 (PMC4908804; doi:10.1186/s13643-016-0279-4)
Supplement: Additional file 1: — Search strategies. The data provided shows the comprehensive search strategy for each database. (DOCX 15.5 kb) [file 13643_2016_279_MOESM1_ESM.docx]

Search strategy for MEDLINE

| 1 | organ transplantation/ or heart transplantation/ or kidney transplantation/ or liver transplantation/ or lung transplantation/ |
| --- | --- |
| 2 | ((organ or heart or kidney or renal or liver or lung) adj transplant*).tw. |
| 3 | 1 or 2 |
| 4 | "Outcome Assessment (Health Care)"/ |
| 5 | "Process Assessment (Health Care)"/ |
| 6 | "Outcome and Process Assessment (Health Care)"/ |
| 7 | Health Status Indicators/ |
| 8 | "Quality of Life"/ |
| 9 | "Quality of Health Care"/ |
| 10 | Quality Indicators, Health Care/ |
| 11 | Patient satisfaction/ |
| 12 | Health Status/ |
| 13 | patient outcome assessment/ |
| 14 | Quality Assurance, Health Care/ |
| 15 | Quality Improvement/ |
| 16 | quality.tw. |
| 17 | patient satisfaction.tw. |
| 18 | (clinical adj2 indicator).tw. |
| 19 | 4 or 5 or 6 or 7 or 8 or 9 or 10 or 11 or 12 or 13 or 14 or 15 or 16 or 17 or 18 |
| **20** | **3 and 19** |

Search strategy for Embase

| 1 | *organ transplantation/ or *heart transplantation/ or *kidney transplantation/ or *liver transplantation/ or *lung transplantation/ |
| --- | --- |
| 2 | ((organ or heart or kidney or renal or liver or lung) adj transplant*).ti. |
| 3 | 1 or 2 |
| 4 | health status indicator/ |
| 5 | *outcome assessment/ |
| 6 | *health care quality/ |
| 7 | *"quality of life"/ or *"quality of life assessment"/ |
| 8 | patient satisfaction/ |
| 9 | quality.tw. |
| 10 | *outcomes research/ |
| 11 | health services research/ |
| 12 | patient satisfaction.tw. |
| 13 | health status/ |
| 14 | quality control/ |
| 15 | total quality management/ |
| 16 | (outcome adj2 assess*).tw. |
| 17 | (process adj2 assessment).tw. |
| 18 | (clinical adj2 indicator).tw. |
| 19 | 4 or 5 or 6 or 7 or 8 or 9 or 10 or 11 or 12 or 13 or 14 or 15 or 16 or 17 or 18 |
| 20 | 3 and 19 |

Search strategy for EMB Reviews – Cochrane Central Register of Controlled Trials and Cochrane Database of Systematic Reviews

1. organ transplantation/ or heart transplantation/ or kidney transplantation/ or liver transplantation/ or lung transplantation/

2. ((organ or heart or kidney or renal or liver or lung) adj transplant*).hw.

3. 1 or 2

4. "Outcome Assessment (Health Care)"/

5. "Process Assessment (Health Care)"/

6. "Outcome and Process Assessment (Health Care)"/

7. Health Status Indicators/

8. "Quality of Life"/

9. "Quality of Health Care"/

10. Quality Indicators, Health Care/

11. Patient satisfaction/

12. Health Status/

13. patient outcome assessment/

14. Quality Assurance, Health Care/

15. Quality Improvement/

16. quality.tw,hw.

17. patient satisfaction.tw,hw.

18. (clinical adj2 indicator).tw,hw.

19. 4 or 5 or 6 or 7 or 8 or 9 or 10 or 11 or 12 or 13 or 14 or 15 or 16 or 17 or 18

20. 3 and 19
