# Supplementary material for: Quality metrics in solid organ transplantation: protocol for a systematic scoping review
Source: Syst Rev. 2016 Jun 14;5:99. doi: 10.1186/s13643-016-0279-4 (PMC4908804; doi:10.1186/s13643-016-0279-4)
Supplement: Additional file 3: — The appraisal of candidate measures using the criteria for a good measure. The data provided demonstrates the tool that will be used to determine if a quality metric meets the criteria for a good measure. (DOCX 18.6 kb) [file 13643_2016_279_MOESM3_ESM.docx]

Additional File 3 – Appraisal of Candidate Measures using the Criteria for a Good Measure

Each of the four criteria for a good measure will be asked of each metric. Answers may include yes (+), no (-) and unclear (?). The criteria for a good measure will be interpreted as follows:

1. Easy to define and observe: Objective, well defined events

2a. Important to patients: Quality measurements are tailored to outcomes important to patients

2b. Important to health care providers: Quality measurements are tailored to outcomes important to health care providers and may prompt them to make changes in response to the metric

3. Amenable to change: Will help define areas that are ripe for improvement, particularly where there is substantial variation in a measure.

4. Obtainable from existing or easily collected data: data is collected from existing databases or easily collected from the patient or institution.
